# Genomic Investigation of Infantile and Childhood Epileptic Encephalopathies in Kazakhstan: An Urgent Priority

**DOI:** 10.3389/fneur.2021.639317

**Published:** 2021-06-10

**Authors:** Altynshash Jaxybayeva, Alissa Nauryzbayeva, Assem Khamzina, Meruert Takhanova, Assel Abilhadirova, Anastasia Rybalko, Kymbat Jamanbekova

**Affiliations:** ^1^Department of Neurology, Astana Medical University, Nur-Sultan, Kazakhstan; ^2^Department of Neurology of Early Age, National Research Center for Maternal and Child Health, Nur-Sultan, Kazakhstan; ^3^Department of Early Age Neurology, University Medical Center, National Research Center for Maternal and Child Health, Nur-Sultan, Kazakhstan; ^4^Corporate Fund University Medical Center, National Research Center for Maternal and Child Health, Nur-Sultan, Kazakhstan; ^5^Nazarbayev Intellectual School, Nur-Sultan, Kazakhstan

**Keywords:** infantile, childhood, epileptic encephalopathy, genomic investigation, management

## Abstract

**Objectives:** Infantile and childhood epileptic encephalopathies are a group of severe epilepsies that begin within the first year of life and often portend increased morbidity. Many of them are genetically determined. The medical strategy for their management depends on the genetic cause. There are no facilities for genetic testing of children in Kazakhstan but we have a collection of data with already defined genes responsible for clinical presentations.

**Methods:** We analyzed children with epileptic encephalopathies that began in the first 3 years of life and were accompanied by a delay/arrest of intellectual development, in the absence of structural changes in the brain. Such patients were recommended to undergo genetic testing using epileptic genetic panels in laboratories in different countries.

**Results:** We observed 350 infants with clinical presentation of epileptic encephalopathies. 4.3% of them followed our recommendations and underwent genetic testing privately. In total 12/15 children became eligible for targeted treatment, 3/15 were likely to have non-epileptic stereotypies/movements, 2/15 were unlikely to respond to any therapy and all had a high chance of intellectual disability, behavioral and social communication disorders.

**Conclusion:** The genetic results of 15/350 (4.3% of patients) have demonstrated the potential and enormous impact from gene panel analysis in management of epileptic encephalopathy. Availability of genetic testing within the country will improve management of children with genetic epilepsies and help to create a local database of pathogenic variants.

## Introduction

Infantile and childhood epileptic encephalopathies (ICEE) are a group of epilepsies, estimated to affect ~1.2/1,000 live births (1, 2). They are typified by multiple types of seizures within the first years of life, developmental delay, and resistance to anti-epileptic drugs (AED) (3). In ICEE, not only does epileptic activity contribute to cognitive/behavioral impairments, but they would also be expected from the underlying pathology alone. Expanding knowledge of the geno/phenotypes of genetic epilepsies led to the concept of epileptic and developmental encephalopathies (EDE). In EDE, the developmental impairment occurs as a direct result of the genetic variant, in addition to the effect of excessive epileptic activity. It is estimated that genetic epilepsies include more than 30% of all epilepsy syndromes. Several genetic tests are now available for diagnostic purposes in clinical practice. In particular, next-generation sequencing has proven to be effective in revealing gene variants causing epilepsies in up to a third of the patients (4).

According to the classification of the International League Against Epilepsy (ILAE), ICEE are distinguished separately, on the basis of common electro-clinical characteristics, age of onset, type of seizures, etiology, and prognosis (1, 2). ICEE such as Ohtahara syndrome, early myoclonic encephalopathy, epilepsy of infancy with migratory focal seizures, and West, Dravet, and Lennox–Gastaut syndromes are more common (2). Most ICEE are resistant to pharmacological therapy and may have significant comorbidity, such as developmental delay, developmental arrest, or regression, due to both the presence of epilepsy and the underlying causes of ICEE (e.g., gene variant or structural abnormality) (1, 3).

Following ILAE recommendations, it is necessary to identify the cause, as it can determine comorbidity, therapy, and prognosis (2, 5, 6). In our experience, there is a lack of information regarding prevalence of infantile and childhood epileptic encephalopathies in Eurasia. The underlying etiology of MRI negative cases is rarely identified.

To this date, more than 500 genes are associated with epilepsies (7, 8). Symonds et al. (9) reported seven genes with disease-causing variants, accounting for 80% of ICEE in children under 3 years of age in their series. About 50% of variants were identified in genes coding for sodium and potassium channel components (10). Variants in SCN1A and SLC2A1 were more frequent than those reported in studies conducted in Denmark, California, and Queensland (11–14). A Scottish study has shown the feasibility of conducting genetic testing for children with epilepsy in the first 3 years of life, which has led to an optimization of diagnostic and treatment approaches (9). Molecular diagnosis can influence management and translate into better/specific treatment recommendations (10).

The feasibility of genetic investigation for ICEE and the spectrum of genomic variants identified in Eurasia have not been previously reported. In our study, we aim to show the potential to improve management as well as profile of these investigations.

## Materials and Methods

The study was conducted at the KFUMC National Research Center for Maternal and Child Health (Nur-Sultan, Kazakhstan) with the participation of patients of Children's municipal hospital No. 2 in Nur-Sultan from 2018 to 2020. We analyzed children who had febrile and afebrile seizures that began in the first 3 years of life were accompanied by a delay/arrest of development, in the absence of structural changes in the brain seen on MRI that might explain the epilepsy (as opposed to stigmata of an underlying genetic disorder). The analysis of phenotypic signs and the assumption of the presence of an epileptic syndrome were carried out according to the criteria for the diagnosis of EE of the ILAE revision 2017. Statistical data on the number of children diagnosed with epilepsy in Kazakhstan were taken from the reports of regional coordinators for pediatric neurology in regions and cities of Republican significance as of October 1, 2020.

Family history, birth, onset/type of first seizures, neurological development, the presence of structural changes, features of the electroencephalogram, and response to anticonvulsant therapy were taken into account. Consents were obtained from the parents before performing genetic analysis.

When the phenotype did not correspond to any recognizable syndrome, patients were included in the unclassified group. The assumption that epileptic seizures in children are of a genetic nature occurred in the presence of ICEE, absence of structural changes on MRI of the brain, and/or relevant history/examination. Epileptic genetic panels were performed in laboratories in Russia, Germany and France, since this type of diagnostics is not available in Kazakhstan.

The variants were described as pathogenic, when they were consistent with the phenotype of the epileptic encephalopathy and reported as disease causing by the relevant molecular genetic laboratory (15–23). We did not reports variants with unknown significance.

## Results

The coordinators of pediatric neurology of 3 cities and 14 regions of the Republic of Kazakhstan, as of October 2020, reported that there were 15,769 children (aged 0 to 18 years) diagnosed with epilepsies. Of these, 15,126 children with generalized and focal epilepsies were coded under ICD-10 as G40.1 and G 40.2. Children identified with various epileptic syndromes were coded as G40.4, such as Dravet Syndrome-−245; West syndrome−187; Landau–Kleffner syndrome−33; Lennox–Gastaut syndrome−104; Ohtahara syndrome−8; epilepsy with status epileptics in slow sleep−30; and early myoclonic epilepsy−36.

A total of 643 children were identified with ICEE requiring multiple anticonvulsant therapy, encoded by G 40.4, of whom 361 children were under 5 years of age and 282 children were 5–18 years old. The current population of Kazakhstan, based on the latest United Nations data, is 18,776,707 people including children from 0 to 15 years old−4,087,008. Thus, the prevalence of epileptic encephalopathy per 100,000 population of Kazakhstan is 3.4 and that among the child population from 0 to 15 years old is ~15.7 per 100,000.

During the period from 2018 to 2020, we assessed 350 children with a diagnosis of ICEE, who were assessed at the KFUMC National Research Center for Maternal and Child Health (Nur-Sultan, Kazakhstan) with the participation of patients from Children's Municipal Hospital No. 2 in Nur-Sultan. All were recommended to perform a NGS gene panel analysis. In 15, we identified the cause of their condition. Testing was dependent on the parent's ability to pay for the analysis.

Family history of all were negative.

Three children had a mutation in CDLK5. In all, the onset of seizures occurred in early infancy up to 3 months; in two, there was a temporal relationship to DTP vaccination, and one, with seizure onset on day 29 of life, was jaundiced. Focal, tonic–clonic seizures and infantile spasms were noted. Vigabatrin reduced seizure burden.

MECP2 variants were found in two children. The onset of symptoms was at 8–9 months, with stereotypical movements, generalized tonic–clonic seizures, and delay in intellectual development. Levetiracetam resulted in seizure remission for 6 months in one patient.

A child with a heterozygous variant in the STXBP1 gene had the onset of infantile spasms at the age of 2.5 months, which evolved into focal seizures and were accompanied by a developmental delay. There was some response to vigabatrin.

One case with single focal seizures was associated with a heterozygous microdeletion of the UBE3A gene—Angelman syndrome (15). She had delay in mental and motor development from 3 to 4 months, the presence of chaotic movements in limbs, and microcephaly.

The girl with a heterozygous variant in the PCDH19 gene developed seizures aged 1.5 years with a positive response to sodium valproate and topiramate.

A boy with a FOLR1 variant had seizures on awakening at onset. At 1.5 years, he had recurrent tonic seizures, which evolved into generalized tonic–clonic seizures mainly during sleep. At 4.5 years, there was motor and cognitive regression, foot tapping stereotypies, and loss of self-care skills.

The child with a PNPO variant differed from those presented above with the development of status epileptics from the 6th day of life. The seizures were stopped by propofol until an infusion of pyridoxine was applied for diagnostic purposes, after which the child became more active and spontaneous physical activity appeared.

Two boys with heterozygous variants in SCN1A had fever-induced prolonged hemiclonic seizures against from 6 months of age and delay in intellectual development. Resistance to therapy, including stiripentol, was noted.

Table of identified phenotypes and genotypes (Tables 1, 2).

**Table 1:** Identification of phenotypes and genotypes.

	**SCN1A**	**CDLK5**	**MECP2**	**STXBP1**	**UBE3A**
Number of patients	2	3	2	1	2
Gender	Males	Females	Females	Male	Females
Age of disease onset	6 months	Before 3 months	8–9 months	2.5 months	3–6 months
Age of genetical diagnosis	12 months 15 months	40 months 13 months 9 months	19 months 44 months	5 months	8 months 16 months
Type of seizures	Hemiclonias, febrile seizures, status epilepticus tonic clonic	Focal; generalized tonic-clonic; infantile spasms	Generalized tonic-clonic Non-epileptic stereotyped movements	Infantile spasms	Focal seizures and chaotic movements in the limbs
EEG	Acute slow wave with emphasis in the frontal/central-parietal and temporal leads bilaterally	Hypsarrhythmia variants with “burst -suppression”	Slowing of main activity; Multifocal epileptiform activity with secondary bilateral synchronization in the form of peak/polypeak/sharp-slow wave complexes	Slow-wave type of EEG, at deepening of sleep weakly expressed “burst -suppression” areas appear	High-amplitude disorganized slow waves, constant continuous deceleration
Cognitive status	Speech delay	Intellectual delay	Regression of cognitive development	Speech delay	Intellectual delay
MRI	Normal	Normal	Normal	Normal	Normal
Response to therapy	Stiripentol resistance, good response to hydrocortisone, Topiramate, Valproate led to increased seizure frequency, clonazepam was not effective	Positive response to vigabatrin	Reduction of seizures when taking levetiracetam, topiramate	Positive response to vigabatrin	Positive response to levetiracetam
	**FOXG1**	**ATP7A**	**PCDH19**	**FOLR1**	**PNPO**
Number of patients	1	1	1	1	1
Gender	Female	Male	Female	Male	Male
Age of disease onset	2 months	2 months	1.5 years	1.5 years	6th day of life
Age of genetical diagnosis	24 months	11 months	36 months	8 year	75 days of life
Type of seizures	Apnoea; fading; focal seizures with eyes upward and arms extended to the sides; face palor	Clonic seizures with eyes deviation	Tonic; then with a predominance of the focal component (fading with head rotation)	Focal; then tonic-clonic. Subsequently–the development of non-epileptic stereotypies	Status epilepticus
EEG	Slow wave EEG; periodic secondary generalized epileptiform activity in the form of diffuse burst of polymorphic complexes spike/polyspike/sharp-slow waves with a focus in the right central temporal leads. Burst suppression.	In wakefulness and sleep, status epilepticus. Epileptiform activity in the form of irregular sharp waves and complexes sharp slow waves	Periodic epileptiform activity with complexes of sharp-slow waves	Bursts of spike/polyspike/sharp-slow waves followed by a short suppression of the electrical activity of the brain	Constant pattern of burst suppression
Cognitive status	Speech delay	Intellectual delayed	Normal	Speech delay	Intellectual delay
MRI	Normal	MR signs of diffuse lesions of the white matter of the brain with the formation of cysts	Normal	leukoencephalopathy	Hypoplasia of the temporal lobes and corpus callosum
Response to therapy	Poor response to levetiracetam and valproate	Good response to IV diazepam; partial response to topiramate; no response to valproate	Good response to topiramate, valproate led to increased seizure frequency	Levetiracetam, lamotrigine, phenobarbital, clonazepam, pregabalin–not efficacious. Response to calcium folinate therapy	The seizures were stopped by the introduction of propofol until an infusion of pyridoxine was made for diagnostic purposes

**Table 2:** Description of disease causing variants at 15 patients.

**No**	**Gender**	**Age of genetic analysis**	**Disease causing variants**
**SCN1A gene**			
1	Male	15 months	c.4852+2T>A; p.? (het.), splice effect
2	Male	12 months	c.4251+2T>C (het), splice effect
**No**	**Gender**	**Age**	**Disease causing variants**
**CDKL5 gene**			
1	Female	3.4 years	chrX:18627006TC>T (het.) c.2022delC p.Phe675fs
2	Female	13 months	chrX:18622134GAAAGCTTCCT>G c.1094_1103delGCTTCCTAAA p.Ser365fs
3	Female	9 months	chrX:18646628CCT>C (het.) c.2635_2636delCT p.Leu879fs
**No**	**Gender**	**Age**	**Disease causing variants**
**MECP2 gene**			
1	Female	1.7 years	chrX:153296090c>T (hem.) p.E409K
2	Female	3.8 years	c.808C>T, (het.) p.(Arg270*)
**FOXG1 gene**			
1	Female	2 years	heterozyg c 797 T>A; p.1266N
**STXBP1 gene**			
1	Male	5 months	chr9:130428485G>A (het.) c.704G>A p.Arg235Gln
**UBE3A gene**			
1	Female	8 months	Heterozygous microdeletion of gene UBE3A region 15q11.2 (SNRPN-u5–0.58; SNRPN-CpG isl−0.55; UBE3A-10–0.55; UBE3A-1–0.55)
2	Female	16 months	microdeletion of gene UBE3A region 15q11.2 hg18 loc: 15-023.201527 heterozyg
**PCDH19 gene**			
1	Female	3 years	chrX:99662504C>CG rs758946412 (het.) c.1091dupC p.Tyr366fs. 1 exon
**ATP7A gene**			
1	Male	11 months	chrX:77264673C>G (hem.) ATP7A NM_000052.6 c.1782C>G p.Tyr594*
**FOLR1 gene**			
1	Male	8 years	chr11:71906498C>T (het.) FOLR1 c.352C>T p.Gln118* and chr11:71906764 T>G c.466T>G p.Trp156Gly
**PNPO gene**			
1	Male	2.5 months	PNPO c.673C>T; p.Arg225Cys (homo)

## Discussion

There is a lack of data available about the normal genome sequence/variants in the Eurasian population and prevalence of ICEE in neighboring countries, e.g., Uzbekistan, Kyrgyzstan, and Russia. In Kazakhstan, we identified 643 children with possible genetic epilepsies, but genome sequencing was not available for all. The prevalence of epileptic encephalopathy per 100,000 population of Kazakhstan is 3.4, and in the group 0–15 years, the prevalence is approximately 15.7/100,000 population.

We have identified a limited number of the children (4.3%) using widely dispersed laboratories. This has led to costs for families as well as concern as to the accuracy and interpretation of results. We have demonstrated a very high yield in terms of results that have profound implications for the families.

In all cases, the variants allowed us to make more accurate and therapeutically useful interventions. We noted a high rate of diagnoses for which disease modification therapy was available. These include variants in PNPO, FOLR1, ATP7A, and SCN1A for which precision disease-modifying therapy (DMT) is easily available with pyridoxine, leucovorin calcium, copper (if early), and fenfluramine, respectively (10, 16, 23–26).

An even higher number of patients had genotypes that would suggest the evidence-based trial of therapies including the DMT, but also for SCN1A/PCDH19 (cannabinoids, clobazam, and stiripentol), UBE3A (antimyoclonic therapies including sodium valproate and clonazepam), and CDKL5/MECP2 (the ketogenic diet) (18, 27, 28).

Conversely, the diagnosis contraindicated certain therapies in SCN1A/PCDH19/UBE3A—promyoclonic drugs such as vigabatrin and carbamazepine. We noted that some genes had no evidence-based therapy that would give a significant chance of seizure improvement. Although distressing to families, this knowledge allows us to focus on non-pharmacological supportive therapies (these included FOXG1 and STXBP1) (29).

In some, the high rate of non-epileptic paroxysmal movements (MECP2, FOXG1) is likely to be informative and reduce the risk of inappropriate treatment of non-epileptic phenomena with anti-epileptic drugs (30).

In total, 12/15 children became eligible for targeted or DMT, 3/15 were likely to have non-epileptic stereotypies/movements, at least 2/15 were unlikely to respond to any therapy, and all had a high chance of intellectual disability and behavioral and social communication disorders.

The phenotype and genotype were very useful in the diagnosis and choice of management. If the phenotype can be determined using clinical data, then genetic testing is necessary. If the phenotype cannot be determined due to the spectrum of clinical manifestations of different syndromes, then testing a panel of genes becomes appropriate.

A Scottish population study of children with epilepsy showed that monogenic epilepsy is more common than previously thought, at 1 in 2,000 live births (9). Most of the cases were *de novo* dominant variants and is likely similar in other populations. The use of gene panels for epilepsy increases the likelihood of correct diagnosis (4, 10, 31). The rate of diagnosis using targeted sequencing of a single gene was 15.4%; this rate increased to 46.2% using specialized genetic panels—a cost-effective alternative to Sanger sequencing (4, 8, 9, 29, 32).

Genetic testing/whole exome sequencing are not currently available in most Eurasian centers, but when they become more widely available, this will lead to improved therapeutic outcomes (29).

If we take into account the number of newborns in the Republic of Kazakhstan in 2019, which amounted to 402,310 according to the statistical agency (www.stat.gov.kz), then using the data obtained on the frequency of monogenic epilepsy in the population (1 in 2,000), we can expect up to 200 new cases of ICEE age per year.

We have demonstrated the potential and enormous impact of positive results from gene panel analysis in ICEE in Eurasia. Epilepsy gene panel analysis should be available within Eurasia and in particular should allow clinical–laboratory discussion of variants to confirm genotype/phenotype correlation accurately. It will allow us to design region-specific gene panels and coverage and to establish the inheritance of ICEE. More importantly, country analysis would allow standardization and quality control of both the sequencing and interpretation of variants.

### Limitation of the Study

This is the first data about genetic assessment of children with ICEE from the Eurasia region. Genetic analyses were made in different laboratories and were dependent on parents' abilities to arrange and pay. We had difficulties in analyzing variants with unknown significance and limited information about gene panels and coverage. It was not always possible to establish inheritance due to additional expenses. The lack of information about Eurasian genotypes/referring only to European data could have led to both false-positive and -negative results. As the analyses were done outside Kazakhstan, we were unable to confirm the standards to which the sequencing was performed and reported.

## Data Availability Statement

The raw data supporting the conclusions of this article will be made available by the authors, without undue reservation.

## Author Contributions

AJ wrote an article, analyzed data, took part at selection, and assesment of kids as a senior counsaltant neurologist of the group, has equal contribution and first authorship. AK a treating doctor to the patients, and took part at selecting of medical information, has an equal contribution and second authorship. KJ provided with all references and full texts articles mentioned at the manuscript. AR technical support during collection and analyzing the data. AA also a treating doctor for the selecting patients, has an equal contribution and second authorship. MT took part at selecting information as a treating doctor for the patients, has an equal contribution and second authorship. AN took part at selecting medical information as a treating doctor for the patients, also has equal contribution and second authorship. All authors contributed to the article and approved the submitted version.

## Conflict of Interest

The authors declare that the research was conducted in the absence of any commercial or financial relationships that could be construed as a potential conflict of interest.
